# Clinical Lecture

**Published:** 1839-05-18

**Authors:** 


### CLINICAL LECTURE.

[From a lecture by Dr. Wm. Harris, delivered at the Philadelphia Medical Institute, we report the following interesting case.—Eds.]

### THE CLITORIS.

The clitoris is a pendant body, situated in front of the middle of the symphysis pubis, about an inch below the superior commissure of the vulva. It resembles, in many respects, the male penis, consisting of a body an inch long, and two crura, each of which is, also, an inch long. These crura, like the crura of the male penis, are attached to the inside of the ischio-pubic rami, at their junction. The clitoris has two corpora cavernosa, but no corpus spongiosum. It has, moreover, a fold of skin which hangs in a crescent form over its extremity, which is called the preputium clitoridis, and which causes it to resemble the glans and prepuce of the male penis. It has, however, no real glans, nor is there any canal.

There are various hypotheses with regard to its use. Most authors make it the seat of pleasure during coition, and endow it with a high degree of erotic sensibility.

The clitoris sometimes, though the instances are very rare, becomes *hypertrophied* in so high a degree that, without careful inspection, it could not be distinguished from the male organ, and hence the prevalent belief, among the vulgar, in the existence of hermaphrodites.

An interesting case, of a supposed hermaphrodite, came under my own observation in Chester county, in this state. After the birth of the child, the clitoris being unusually long, there was a regular consultation among a number of gossiping females assembled on the occasion, who, after due inspection, unanimously agreed that it was a hermaphrodite; but as the organs of generation, in their opinion, resembled the female's more than the male's, it was called Elizabeth. As the child advanced in years, the clitoris increased in length more rapidly than the male organ usually does. She grew very robust, assumed a masculine appearance, delighted only in manly sports and the labours of the field. At eighteen years of age, she was nearly six feet high, and of vigorous proportions; her beard began to grow and she reversed the decision of the gossips who attended her birth—and, of her own accord, exchanged her female attire for men's apparel, and her name, which was Elizabeth, for Rees, and declared, in a solemn manner, that if any person from that time forward should dare to call her Elizabeth, she would take his life.

She now abandoned all the duties of a female, and was employed only in the out-door labour of a farm, such as ploughing, sowing, reaping, mowing, chopping wood, &c.

At five and twenty years of age she had never menstruated,—had a very strong beard,—was industrious and enterprising, and had increased rapidly in wealth by farming, and by the purchase and sale of farm stock. She was remarkably athletic, and the terror of the whole neighbourhood in personal conflict. At fisticuffs, she was considered "*a regular bully.*" About this period she married a woman named Elizabeth, whom she treated with some kindness. She had great enjoyment in the married life, and especially in copulation. In the act of coition, she used the clitoris, which was now five or six inches long, as a male organ, and declared that she experienced the most consummate pleasure. There was no semen masculinum ejected, and of course there was no offspring. Indeed, she often told her companion that she was no "child getter," and that if she ever became pregnant, she would put her to death.

This extraordinary female lived until she was sixty years of age, and died in possession of a large estate, which she had accumulated by her industry and enterprise.

I attended her in her last illness, and the day on which she died she directed a faithful old male servant to lay her out after death, and not to allow any other person to be present during that process; not to remove the pantaloons and shirt she then had on, but to put clean ones over them, and to remember that it was her dying request that no examination should be made of her person by any physician. I was, therefore, positively denied the privilege of making a post mortem examination; but from all I could learn from her friends, she differed in her genital organs from those of other females in nothing but her enormously elongated clitoris. Throughout her life she always evacuated her urine as a woman, in a squatting position.

As she never menstruated, it is probable her ovaries were defective. She had the appearance, size, and strength of a large and robust man.

This anomalous case is calculated to strengthen the hypothesis that the clitoris is the seat of erotic pleasure, and to corroborate an opinion I have long entertained, that the female is mainly indebted to menstruation for the delicacy of her beauty and the effeminacy of her character. I do not remember to have seen the remark made by any medical writer; but I have repeatedly observed that females who menstruate with great difficulty, or who do not menstruate at all, are annoyed by strong and unsightly beards, often become fat and gross, and assume a general masculine appearance. Indeed, observation and experience have so connected, in my mind, the female beard with dysmenorrhœa and amenorrhœa, that the presence of mustaches is now presumptive evidence of the existence of one or the other disease. Dysmenorrhœa and amenorrhœa, doubtless, in most cases, arise from defective ovaries, as females afflicted with these diseases very rarely bear children. As the defection of the ovaries, and the suppression of the menses, causes the female to assume the general appearance of the man, so the removal of the testicles in a great measure deprives the man of his beard, and makes him assimilate the female, both in appearance and general effeminacy.

The same thing takes place in animals. If the testicles of the male and the ovaries of the female be removed in early life, they will lose their distinctive character: so much so, that without examining the genital organs, the one could not be distinguished from the other. The *bull* and the *cow* are very different looking animals; but the *spayed heifer* and the *steer* are precisely alike in their size and general appearance.

